# The gut–liver axis in sepsis: interaction mechanisms and therapeutic potential

**DOI:** 10.1186/s13054-022-04090-1

**Published:** 2022-07-13

**Authors:** Xue Zhang, Hong Liu, Kenji Hashimoto, Shiying Yuan, Jiancheng Zhang

**Affiliations:** 1 Department of Critical Care Medicine, Union Hospital, Tongji Medical College, Huazhong University of Science and Technology, Wuhan, 430022 People’s Republic of China; 2 Institute of Anesthesia and Critical Care Medicine, Union Hospital, Tongji Medical College, Huazhong University of Science and Technology, Wuhan, 430022 People’s Republic of China; 3 Division of Clinical Neuroscience, Chiba University Center for Forensic Mental Health, Chiba, 260-8670 Japan

## Abstract

Sepsis is a potentially fatal condition caused by dysregulation of the body's immune response to an infection. Sepsis-induced liver injury is considered a strong independent prognosticator of death in the critical care unit, and there is anatomic and accumulating epidemiologic evidence that demonstrates intimate cross talk between the gut and the liver. Intestinal barrier disruption and gut microbiota dysbiosis during sepsis result in translocation of intestinal pathogen-associated molecular patterns and damage-associated molecular patterns into the liver and systemic circulation. The liver is essential for regulating immune defense during systemic infections via mechanisms such as bacterial clearance, lipopolysaccharide detoxification, cytokine and acute-phase protein release, and inflammation metabolic regulation. When an inappropriate immune response or overwhelming inflammation occurs in the liver, the impaired capacity for pathogen clearance and hepatic metabolic disturbance can result in further impairment of the intestinal barrier and increased disruption of the composition and diversity of the gut microbiota. Therefore, interaction between the gut and liver is a potential therapeutic target. This review outlines the intimate gut–liver cross talk (gut–liver axis) in sepsis.

## Introduction

Sepsis is a life-threatening organ dysfunction caused by a dysregulated host response to infection, with high morbidity and mortality worldwide [1]. Indeed, 2.8 million deaths per year are attributable to sepsis in high-income countries [2]. Sepsis can progress to multiple organ dysfunction syndrome (MODS) [3]. Liver dysfunction in MODS patients is typically associated with significant morbidity, though its exact prevalence remains unknown.

The gut microbiota is recognized as a powerful indicator of disease-related morbidity and progression [4]. For instance, Lachnospiraceae contributes to protecting the intestinal mucosal barrier and offers a microbe-mediated survival advantage in a murine model of sepsis [5]. *Bifidobacterium*, Muribaculaceae, *Parabacteroides distasonis*, and *Alloprevotella* are involved in protection against sepsis-related liver injury in rats [6]. The gut microbiota is key to the development and regulation of the immune system, impacting host susceptibility and response to infection [7]. Intestinal dysbiosis and increased intestinal permeability promote pathogenic microbial overgrowth and translocation of intestinal pathogen-associated molecular patterns (PAMPs) to the lymphatic and portal systems, impairing the body’s defense against infection or injury and aggravating organ damage [8]. Cross talk between the gut and liver is widely acknowledged, as the gut and liver communicate bidirectionally via biliary, portal, and systemic circulation [9]. Moreover, intestinal mucosa and vascular barriers serve as a communication hub between the gut and liver. The liver is crucial for modifying the host defense and controlling inflammation in infection [10]. However, under pathological situations, dysregulated translocation of microbial products initiates inflammation that impairs the function and detoxification capacity of hepatocytes. In general, liver dysfunction, decreased bacterial clearance, and metabolic disorders cause increased dysregulation of the gut microbiota and further breakdown of the intestinal barrier, leading to MODS. Hence, improved knowledge of the gut–liver interaction during sepsis-induced liver damage might contribute to elucidating these complicated disorders and provide insight into novel treatment approaches for sepsis that target underlying mechanisms.

## Intestinal barrier

### Physical barrier

The presence of intestinal epithelial cells (IECs) and mucus components serves as a first line of defense to maintain the intestinal barrier. The epithelial and intestinal vascular barrier just below the mucus is composed of a monolayer of cells [11], and this epithelium monolayer acts as a protective barrier, restricting pathogens, toxins, and antigens of the gut lumen from passing into the mesenteric lymph and circulation [12, 13]. Although the epithelium monolayer serves as a primary physical barrier, the paracellular gap is regulated by several intercellular connections, including apical tight junctions (TJs), lower adherens junctions, and desmosomes [14, 15]. Of note, the TJ structure is critical in providing a physical barrier to prevent luminal inflammatory molecules from entering the circulation [16].

Mucus covers the entire intestinal surface, which is composed of goblet cell-derived mucins (MUCs). The small intestine is covered with a single mucus layer, whereas the large intestine contains two major mucus layers, with the inner dense mucus layer working mainly as a protective shield for the gut owing to its impermeability to luminal bacteria [17, 18]. Mucus supplies carbohydrates for commensal bacteria, inhibits epithelial apoptosis, and increases release of immune cell components by acting as a viscous trap for antimicrobial peptides and immunoglobulins [19–21]. MUC2 is a key element of intestinal mucus layers. Muc2 in mice deficiency exacerbates sublethal doses of lipopolysaccharide (LPS)-induced intestinal bacterial translocation to the liver and increases mortality [22].

Furthermore, the intestinal epithelium generates large amounts of antimicrobial peptides (AMPs) and intestinal alkaline phosphatases (IAPs) [23, 24]. AMPs rapidly kill or inactivate bacteria by secreting lysozyme, regulating downstream innate immune responses, and interfering with bacterial cell wall synthesis, among others, which is a method of immune defense that has evolved over time [23].

IAPs can prevent the follow-up toxicity of newly released LPS [24], regulate HCO_3_^–^ secretion [25], and promote growth of gut commensal bacteria [26]. Notably, IAPs also prevent LPS from triggering toll-like receptor (TLR)-4/myeloid differentiation factor 88 (MyD88)-mediated inflammatory cascades [27, 28]. Recent research indicates that IAPs induce autophagy in IECs and macrophages to exert anti-inflammatory effects in mice [29].

### Immune barrier

The immune cells in the gut form a second line of defense to maintain the intestinal barrier (Table 1). Anatomically, the intestinal canal wall is divided into four layers, including the mucosa, submucosa, muscularis, and serosal layer from the lumen inside to outside. Rich lymphatic follicles are present in the propria layer of the mucosal and submucosal tissues, including isolated and aggregated lymphatic follicles [30, 31]. Considering their distribution in the intestinal wall, gut immune cells mainly include intestinal lymph follicles, interepithelial lymphocytes, and lymphocytes in the propria layer of the mucosa [30, 32, 33]. The intestinal epithelial barrier is protected by local interepithelial lymphocytes, which rapidly activate T helper 1 (Th1) cell cytokine responses aimed at an infected or stressed epithelium [34, 35]. Interestingly, dendritic cells (DCs) inserted into the gut epithelium in vitro can open TJs between IECs and directly take up luminal microorganisms. Furthermore, DCs express TJ proteins to preserve epithelial barrier integrity [36]. The propria layer of the mucosa includes plasmacytoid DCs, innate lymphoid cells, mucosa-associated invariant T cells, and T cells to attack pathogens [34, 37, 38]. Secretory immunoglobulin A (IgA) plays a critical role in the adaptive immunity mediated by lamina-intrinsic plasma cells: It regulates gut microbiota composition, protects the intestinal epithelium from pathogenic microbes, and assists in immune system development [39, 40]. By presenting pathogen antigens to intestinal immune cells, IECs contribute to intestinal adaptive immunity [41].

**Table 1:** Gut immune cells in the intestinal wall

Location	Cell type	Function	Refs.
Mucous layer	Intestinal macrophages	Phagocytosis and degradation of microorganisms and dead tissue cells Producing mediators that drive epithelial cell renewal	[33]
	Dendritic cells	Having the ability to open TJs between epithelial cells and directly take up luminal microorganisms	[36]
	Local interepithelial lymphocytes: conventional and nonconventional TCRαβ^+^ subsets; TCRγδ^+^ subgroups	Guarding the intestinal epithelial barrier Rapidly activating cytolytic and Th1-cell cytokine responses aimed at an infected or stressed epithelium	[34, 35]
	ILC1	Being activated by myeloid-cell-derived IL-12	[37, 38]
	ILC2	Being activated by epithelial-derived cytokines and orchestrate type 2 immunity	
	ILC3	Interacting with cells of both the innate and adaptive immune systems Secreting IL-22 and initiating an antimicrobial program as well as barrier fortification in epithelial cells	
	Intestinal B cells	Producing the SIgA	[40]
	Invariant T cells: MAIT cells	Rapidly producing cytokines and exerting cytolytic activity after activation by cells infected with bacteria, including several enteric species	[34]
	iNKT cells	Producing large amounts of IL-4 and IFN-γ involved in the immune response	[34]
Mucous layer and submucosa	Lymphatic follicles	Involved in the local immune response, namely through collaboration with epithelium to effectively localize entry of foreign materials to sites where antigens and microorganisms can be immediately endocytosed, processed, and presented for primary or memory immune responses without the need for systemic involvement	[31]

IFN, interferon; IL, interleukin; ILC, innate lymphoid cell; iNKT, invariant natural killer T; MAIT, mucosal-associated invariant T; TCRαβ^+^, αβ T cell receptor^+^; TCRγδ^+^, γδ T cell receptor^+^; Th1, T helper 1; TJs, tight junctions; SIgA, secretory immunoglobulin A

Membranous cells are specialized epithelial antigen-presenting cells dispersed throughout the follicle-associated epithelium and are critical for antigen-specific IgA production. These gut-associated immune mechanisms protect the systemic circulation from the harmful effects of intestinal pathogens.

### Commensal microbiota in intestines

Human microorganisms are diverse, with an estimated 100 trillion microorganisms composed of between 500 and 1000 distinct bacterial species [42, 43]. The majority have been verified to be composed of five phyla, mainly Firmicutes (79.4%) and Bacteroidetes (16.9%) [44]. Normally, the commensal microbiota influences the intestinal environment, limiting the growth of invasive pathogens. For instance, microbial-derived metabolites inhibit the growth of *Escherichia coli* O157 in vitro [45]. Commensal bacteria also produce bacteriocins that are mainly produced by Firmicutes to fight against invading pathogens [46]. Other bacteria, including Proteobacteria, Bacteroidetes, and Actinobacteria, also encode various bacteriocins. Furthermore, several bacteriocin-producing commensal bacteria (*Bifidobacterium* and *Lactobacillus*), known as probiotics, are used to promote gut health [47]. The distribution, adaptability, and function of the microbial community throughout the gastrointestinal tract are matched with different hospitable environmental conditions, allowing mutual benefits between the host and commensal microbiota.

The stomach of healthy adults has the fewest bacteria, namely *Lactobacillus* and *Helicobacter* species [48], whereas the duodenal microbiota is dominated by Firmicutes and Actinobacteria [49]*.* Firmicutes continues to dominate (43%) in the ileum and colon, but the abundance of Proteobacteria and Bacteroides steadily rises in the ileum and colon [50]. Compared to the small intestine, the large intestine shows greater microbial variety, though the colon tends to be occupied by two significant phyla (Bacteroidetes at 50% and Firmicutes at 45%) [51]*.* The gut microbial community contributes to health by reinforcing the intestinal barrier [52], and commensal bacteria protect against pathogens by competing for nutrients and space [23]. For instance, commensal Enterobacteriaceae fight against *Salmonella* colonization by competing for oxygen in mice [53]. Furthermore, commensal bacteria activate host pattern recognition receptors to enhance production of AMPs and MUCs [18, 23]. They also induce IgA secretion by providing modest levels of immunological stimulation, therefore establishing a basic immune adaptation that plays a crucial role in enabling the host and microbial community to live in homeostasis. Gut microbiota-generated short-chain fatty acids serve as an energy source for commensal bacteria and defend against LPS-induced intestinal barrier disturbance [54]. Overall, microbiota dysbiosis is considered a major contributor to various diseases [52].

## The gut–liver axis

### The physiological “gut–liver axis”

**Fig. 1:**
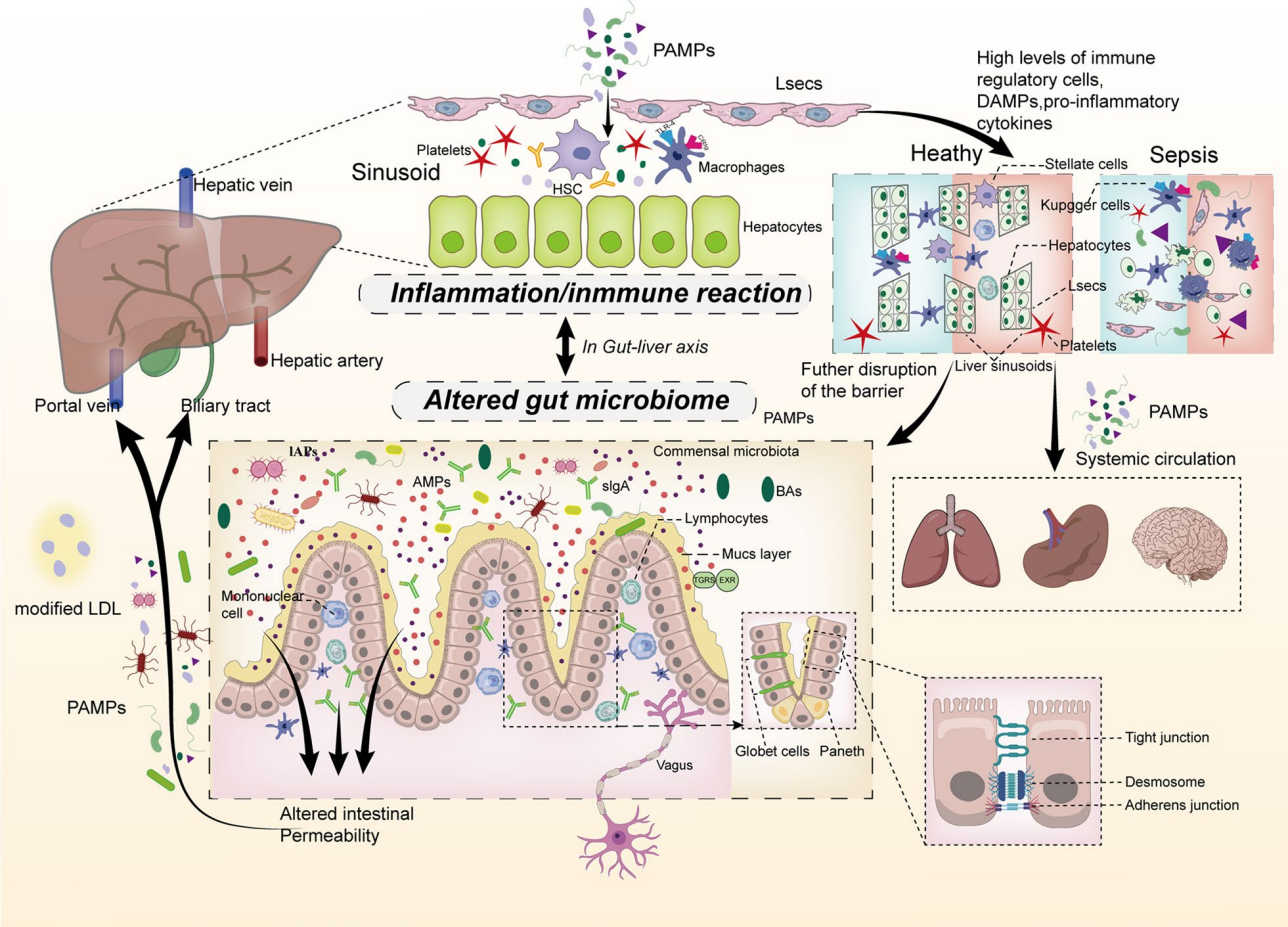
During sepsis, several mechanisms contribute to disruption of the gut barrier, including IEC apoptosis, alteration of the mucus layer, and disruption of intercellular junctions, resulting in translocation of intestinal PAMPs into the liver via the lymphatic vessels, portal circulation, or biliary tract. The liver is essential to the regulation of immune defense, with effector cells such as LSECs, macrophages, stellate cells, and hepatocytes immediately identifying and engaging pathogens, clearing bacteria, and releasing cytokines. When an inappropriate immune response or overwhelming inflammation occurs with high levels of DAMPs and proinflammatory cytokine production in the liver, the normal structure of the hepatic sinus is disrupted, and such cells are damaged through apoptosis and autophagy, leading to bacterial clearance dysfunction and metabolic disorders. As a result, the gut barrier is further damaged, gut microbiota dysbiosis is exacerbated, and distal organs are injured due to the spread of PAMPs and DAMPs and systemic inflammation. IECs, intestinal epithelial cells; DAMPs, damage-associated molecular patterns; LSECs, liver sinusoidal endothelium; PAMPs, pathogen-associated molecular patterns

The liver is the largest gland, secreting bile acids that are ultimately discharged into the small intestine [55], and is supplied by both arterial and venous blood that mix and bathe the various liver structures and cells [56]. The oxygen-rich arterial blood enters the liver via the hepatic artery, but it is a minor blood supply for this organ. Instead, the portal vein, entering the liver with rich nutrients and pathogen-derived molecules such as LPS, is the major blood supply for the liver [57]. Normally, the liver communicates with the gut and its microbiota through the biliary system and systemic circulation (Fig. 1). The bidirectional association of the gut and its microbiota with the liver is well known as the physiological “gut–liver axis” [58].

### Immunological defense of the liver

The liver is also a key immune tissue, with liver sinusoidal endothelial cells (LSECs), macrophages, stellate cells, lymphocytes, and biliary cells comprising the majority of nonparenchymal cells [59–62]. LSECs constitute the inner layer of the hepatic sinus vascular or capillary bed, where nutrient-rich hepatic portal vein blood and oxygen-rich hepatic artery blood mix, contributing to immune surveillance by detecting and capturing pathogens and possibly even presenting antigens and removing macromolecular waste products from the blood [63].

Kupffer cells (KCs) constitute the majority of hepatic macrophages in a healthy liver. The yolk sac-derived colony-stimulating factor 1 receptor-positive erythro-myeloid progenitors and bone marrow-derived monocytes are both sources of KCs [64, 65]. The human liver contains two distinct macrophage subtypes: CD68^+^ macrophage receptor with collagen structure (MARCO)^+^ macrophages, which contribute to the maintenance of immunological tolerance and inflammation suppression, and CD68^+^ MARCO^–^ macrophages, which are more proinflammatory [66, 67].

## Effects of sepsis on the intestinal barrier and microbiota

Inflammation significantly contributes to the pathogenesis of intestinal damage in sepsis [68]. Sepsis affects expression of claudins, junctional adhesion molecule A, occludin, and zonula occludens-1 and activates myosin light-chain kinase, modulating intestinal permeability [69–71]. Owing to activation of TLR4 expressed on intestinal stem cells induced by PAMPs, such as LPS, sepsis may directly affect intestinal stem cell growth and apoptotic death [72, 73]. Intestinal hyperpermeability is strongly associated with dysregulated IEC apoptosis [74], causing changes to the mucus layer, such as decreased thickness, reduced luminal coverage, and poor adhesion [75]. Similarly, decreased villous length is associated with an increase in intestinal permeability and IEC apoptosis [76]. Overexpression of Bcl-2 improves survival by TJ alterations in transgenic mice with the occurrence of hyperpermeability following sepsis [77].

Disruption of intestinal physiology is followed by microbiota dysbiosis in septic patients [78]. In critically ill patients with sepsis, the diversity of the intestinal microbiota declines, and its composition becomes dominated by multidrug-resistant bacteria [79]. Broad-spectrum antibiotic treatment can significantly alter the gut microbiota of severely ill patients [80, 81]. For example, fluoroquinolones increase *Lactococcus* and *Pediococcus* but decrease *Escherichia/Shigella* and *Desulfovibrio* in critically ill patients [82].

## The gut–liver axis in sepsis

### Specific role of lipopolysaccharides

The human gut is a reservoir of ≥ 1 g of LPS from an estimated 100 trillion microorganisms, and endotoxin can be detected even in healthy human plasma [83]. Depending on its size, an LPS molecule passes through multiple alternative paths while crossing the small intestine, which include the paracellular pathway, clathrin-mediated endocytosis [84], micropinocytosis and lipid raft-mediated endocytosis [85], goblet cell-associated antigen passages [86], and the chylomicron pathway [87]. In the colon, LPS can be transported by clathrin-mediated or vesicle-mediated protein transport pathways [88]. When the components of the gut barrier are impacted by inflammation in sepsis, as noted in dysregulated IEC apoptosis, these defense luminal mechanisms that help prevent a large number of LPS transfers to the systemic circulation gradually fail [40], causing transmigration of LPS to multiple organs and triggering uncontrolled immunoinflammatory responses [89].

Typically, intestinal LPS passing through the portal vein is processed and detoxified by the liver. Detoxification of LPS, which is phagocytosed by scavenger receptor (SR)-mediated uptake in the liver, is carried out by acylhydrolase (AOAH) and alkaline phosphatase [90, 91]. AOAH selectively removes secondary fatty acyl chains attached to the primary chain in the lipid A fraction, playing an important role in controlling LPS toxicity [92].

**Fig. 2:**
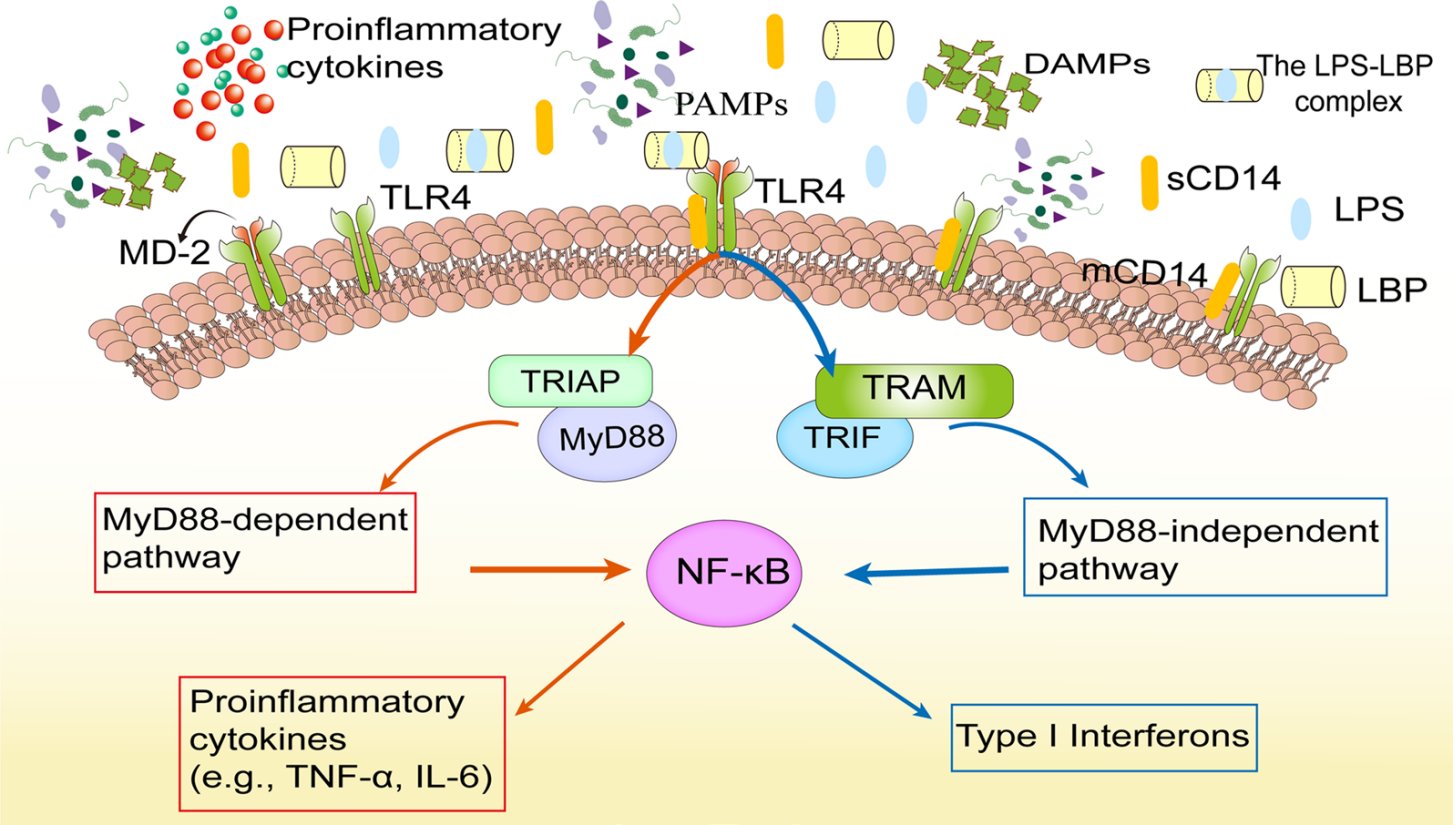
An overview of the signaling pathway of TLR4 activated by LPS. LPS recognition, as facilitated by LBP and CD14, is mediated by TLR4 and the MD-2 receptor complex. Activation of TLR4 signaling, classified into MyD88 dependent, MyD88-independent, and TRIF-dependent pathways, mediates activation of proinflammatory cytokines (TNF-α, IL-6, etc.) and type I interferon genes. IL, interleukin; LBP, LPS-binding protein; LPS, lipopolysaccharide; MD-2, myeloid differentiation-2; MyD88, myeloid differentiation factor 88; TLR4, toll-like receptor 4; TRIF, Toll/IL-1R domain-containing adaptor-inducing IFN-β; TNF, tumor necrosis factor

Additionally, plasma contains some LPS-binding proteins with varying affinities, including LPS-binding protein (LBP), CD14, bactericidal/permeability-increasing protein (BPI), and lipoproteins engaged in LPS detoxification. LBP is primarily produced by IECs and hepatocytes in the acute phase of sepsis in mice [93]. It binds specifically to lipid A of LPS, forming the LPS-LBP complex; this facilitates LPS transfer and binding with membrane CD14 on the surface of monocytes/macrophages and neutrophils, which is finally recognized by TLR4 and myeloid differentiation-2, promoting an inflammatory response cascade [94]. Activation of TLR4 signaling, classified as MyD88 dependent, MyD88-independent, and Toll/IL-1R domain-containing adaptor-inducing interferon (IFN)-β-dependent pathways, leads to markedly increased cytokines and cell damage [95, 96] (Fig. 2). However, high plasma concentrations of LBP and soluble CD14 may help limit deleterious systemic responses to LPS [97]. BPI is expressed in the intestinal epithelium, and changes in potassium levels in damaged cells can act as damage-associated molecular patterns (DAMPs) to promote BPI expression in murine intestinal epithelium [98].

### Effects of gut dysfunction on the liver

After sepsis along with gut dysfunction, PAMPs and DAMPs from the intestine travel through the portal circulation or biliary tract to the liver [58]. In the hepatic vasculature, effector cells immediately engage circulating pathogens or identify PAMPs, forming a network of immunological sentinels [99–103]. Furthermore, TLRs on macrophages and other cell types in the liver are activated by endogenous components from dying host cells, known as DAMPs [95, 102, 104]. NLRs and the RNA-helicase family also recognize pathogens in the cell cytoplasm [102, 105–107]. Gut-derived PAMPs and DAMPs may be a key trigger [108], leading to an inappropriate immune response or overwhelming inflammation, impaired clearance of hepatic pathogenic bacteria, and metabolic disorders [109, 110] (Table 2).

**Table 2:** Roles of gut-derived PAMPs and DAMPs in the liver after sepsis

Components	Roles	Refs.
LPS	Passing through the intestine to reach the IECs via multiple alternative paths Detoxification of LPS by the liver and LPS-binding proteins in plasma Activation of TLR4 signaling Plasma lipoproteins neutralize LPS and accelerate LPS clearance	[84–87, 90, 93, 95]
LSECs	Detecting and capturing pathogens, presenting antigens Contributing to migration of leukocytes to inflammatory sites	[65, 115]
Macrophages		[64, 100–103, 105, 125, 127, 140]
KCs	Constituting the majority of hepatic macrophages in a healthy liver	
Macrophage polarization	M1-like macrophages, as triggered by TLR ligands and IFN-γ, produce proinflammatory cytokines (IL-1β, TNF, IL-6, etc.) M2-like macrophages, as activated by IL-4/IL-13, IL-10, IL-1 receptor antagonist, are critical for anti-inflammatory effects and repairing tissue damage	
Assembly of inflammasomes	NLRP3 and AIM2 inflammasomes cause detrimental inflammation	
Macrophage autophagy	Alleviating hepatic inflammation	
KCs-platelet interaction	Platelet recruitment and limiting bacterial infection in sepsis	
Neutrophils	Migrating to liver sinusoids and releasing NETs to collect and remove bacteria	[143, 144]
NK cells and NKT cells	Contributing to antibacterial defense	[148, 149]
Hepatocytes	Hepatocyte dysfunction and abnormal lipid metabolism Alterations of BA metabolism Causing excretory liver dysfunction	[108–110]
Vagal nerve	Serving as the primary sensory and efferent nerve in the digestive system; among the organs within the abdominal cavity, the liver is a major target of the vagus nerve Stimulation of the efferent arm of vagal circuits can control release of proinflammatory cytokines and promote immune cell activation and differentiation toward a pro-regenerative phenotype Vagus nerve signaling is a critical component of the cholinergic anti-inflammatory pathway	[60–62]

AIM2, absent in melanoma 2; BAs, bile acids; DAMPs, damage-associated molecular patterns; IECs, intestinal epithelial cells; IFN, interferon; IL, interleukin; KCs, Kupffer cells; LPS, lipopolysaccharide; LSECs, liver sinusoidal endothelium; NETs, neutrophil extracellular traps; NK, natural killer; NKT, natural killer T; NLRP3, nucleotide-binding oligomerization domain (NOD)-like receptor protein 3; PAMPs, pathogen-associated molecular patterns; TLR, toll-like receptor; TNF, tumor necrosis factor

#### Liver sinusoidal endothelial cells

Because LSECs lack a basal lamina or septum underneath the endothelium, they provide communication areas known as fenestrae between sinusoidal blood and the subendothelial space, facilitating interchange of substrates between the blood and nearby stellate cells and hepatocytes and regulating lipoprotein traffic in the latter [63, 111]. Blood pressure and toxin levels affect the diameter of LSEC fenestrae in mice [112, 113]. Adhesion molecules of the LSEC immunoglobulin superfamily play crucial roles in leukocyte migration to inflammatory sites [114, 115]. Normal LSECs very faintly express vascular cell adhesion molecule-1, but its expression increases markedly under inflammatory stimulation in rats [116].

LSECs have an extremely high endocytotic capacity that is facilitated by SRs and lysosomal activity, which aids internalization and catabolism of several waste substances and small colloidal particles [117–119]. For instance, SR-A type 1/1.1 binds to various macromolecules, such as LPS and lipoteichoic acid (LTA), in mice [120, 121]. Seven TLRs are now being studied in LSECs [122]. TLR-9 expressed on murine LSECs recognizes and binds unmethylated CpG motifs that are abundant in bacterial DNA to activate nuclear factor kappa-B (NF-κB) and stimulates production of IL-1β and IL-6 [123]. In addition, LSECs initiate antiviral and proinflammatory responses via TLR3 and adaptive responses via TLR1, TLR6, and TLR8 [124]. Hence, LSECs form an anatomical sieve in the liver, continually monitoring antigens such as PAMPs and DAMPs, with proper tolerance, to maintain hepatic immune homeostasis.

#### Macrophages

**Fig. 3:**
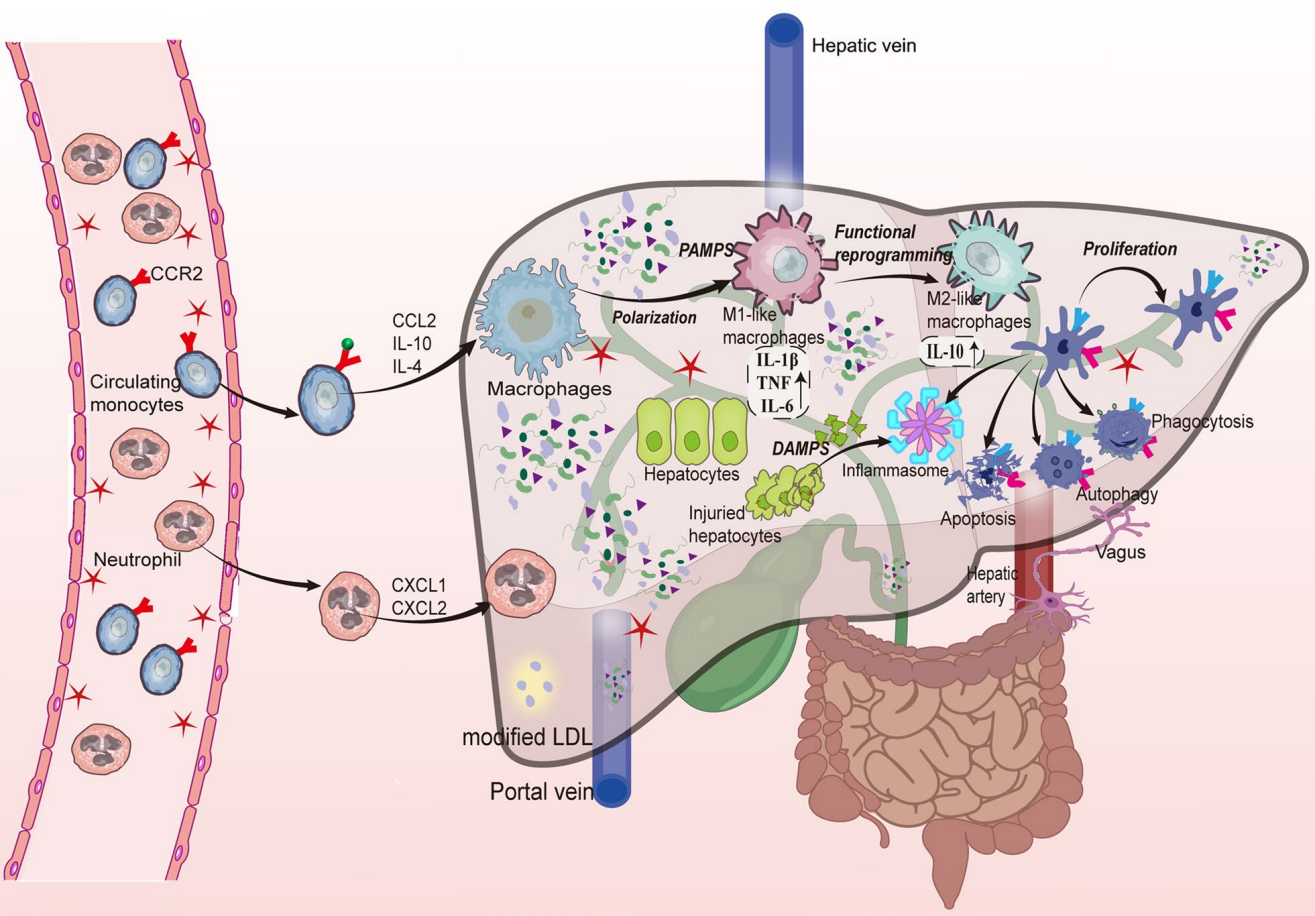
When gut-derived PAMPs are exposed to hepatic macrophages, the macrophages are polarized and form large numbers of M1-like macrophages that mainly produce proinflammatory cytokines such as IL-1β, TNF, and IL-6; some M2-like macrophages typically produce IL-10 and play a role in anti-inflammatory reactions. Inflammasomes are activated in hepatic macrophages and in response to pathogen infections and tissue injury. Moreover, neutrophils are attracted to the liver by chemotactic factors, such as CXCL1 and CXCL2 derived from KCs, and released NETs participate in removal of pathogens and toxins. Platelet recruitment is also critical for limiting bacterial infection, and platelets that interact with KCs play a crucial role in fighting against bacterial infection. However, when an inappropriate immune response or overwhelming inflammation occurs with high levels of DAMP formation and proinflammatory cytokine production in the liver, notable hepatocyte injury, macrophage autophagy, and apoptosis occur. Hepatic macrophages are supplemented by KC proliferation and circulating monocyte recruitment and differentiation. CXCL, chemokine (C-X-C motif) ligand; DAMPs, damage-associated molecular patterns; IL, interleukin; KCs, Kupffer cells; NETs, neutrophil extracellular traps; PAMPs, pathogen-associated molecular patterns; TNF, tumor necrosis factor

Macrophages exhibit variable phenotypes, including M1-like macrophages that are conventionally triggered by TLR ligands and IFN-γ and M2-like macrophages activated by IL-4/IL-13 in addition to various states in between [125–127]. Proinflammatory cytokines are markedly produced by the M1 phenotype, which also generates reactive nitrogen and oxygen intermediates, promotes the Th1 immune response, and plays a significant role in microbicidal and tumoricidal activities [125, 128]. Th2-biased responses are linked to the M2 phenotype, which is typically associated with production of IL-10 and IL-1 receptor antagonists [129, 130]. When macrophages are exposed to bacterial products from the gut in septic mice, they become polarized to the M1 phenotype and participate in the immunoinflammatory response [131]. Sepsis-induced acute liver injury in rats is attenuated by M2-like macrophages, presumably via upregulation of IL-10 expression and suppression of TNF production [132]. Both enhancement of M2-like macrophage polarization and suppression of M1-like macrophage polarization protect the liver from excessive inflammation-induced injury [133, 134] (Fig. 3). Lethal sepsis in mice can be ameliorated by neochromine S5, particularly due to its modulation of M1-like macrophages [135].

Moreover, there are some special roles of KCs in defense against pathogens. For one, complement receptor of the immunoglobulin superfamily (CRIg), expressed on subsets of resident macrophages in various tissues and especially on liver KCs, recognizes activated forms of the complement component C3, playing a significant role in removing additional particles and large complexes from the circulation and in effectively clearing opsonized infectious pathogens [136–138]. *CRIg* gene deficiency in mice markedly lowers KC uptake of *Staphylococcus aureus* and *Listeria monocytogenes* [136]. CRIg is also involved in direct capture of gram-positive bacteria from the circulation by binding to LTA in mice [139]. Bacteria such as *Bacillus cereus* and methicillin-resistant *Staphylococcus aureus* are rapidly captured by KCs in mice and trigger platelets to change their adhesion mode to sustain glycoprotein IIb-mediated adhesion on the KC surface to encapsulate microorganisms, preventing pathogen-induced endothelial permeability and liver damage [140]. However, when addressing macrophage-platelet interactions, it is important to consider immunological repercussions and potential complications such as thrombosis [141, 142]

#### Neutrophils, NK cells, and natural killer T (NKT) cells

Buildup of activated neutrophils in the liver microcirculation may lead to immune-mediated damage [143]. Neutrophil extracellular traps (NETs) are released by neutrophils during sepsis when they migrate to liver sinusoids [144, 145]. LPS or sepsis triggers intravascular NET development mediated by β2-integrin-dependent platelet–neutrophil interactions inside sinusoids in mice [146]. Furthermore, the neutrophil–endothelium interaction and NETs induce microthrombosis and vascular leakage in a rat model of sepsis [147].

NK cells are much more abundant in the liver than in the circulation; NKT cells with several distinct subsets contribute to antibacterial defense and are influenced by the intestinal microbiota. Both NK and NKT cells contribute to the pathophysiology of liver damage [148, 149]. For example, after injection of poly I:C and D-galactosamine in a murine fulminant hepatitis model, NK cells interact with KCs via NK Group 2 member D/ligand recognition, causing severe liver injury [150]. Moreover, when ischemia or toxin-induced injury occurs in the liver, the activated NKT cells predominantly secrete IFN-γ, leading to accumulation of neutrophils and macrophages and the promotion of liver damage in mice [151]. Another study revealed that activation of NKT can kill hepatocytes directly via Fas/FasL pathways in a murine model [152].

## Therapy

### Epithelial barrier-targeting therapy

Due to the role of claudin-2 in gated paracellular channel formation and TJ channel modulation, it may be an ideal therapeutic target for regulating the epithelial barrier [153]. In fact, regulation of TJ channel gating kinetics and protein intermolecular interactions may have therapeutic value in inflammation-associated barrier failure, such as occludin S408 dephosphorylation. However, more studies are needed to identify pharmacological means of modulating gating activity for therapeutic purposes. Moreover, microRNA-155 alleviates inflammation and intestinal barrier dysfunction in septic mice, with a decrease in TNF-α and IL-6 levels via inactivation of NF-κB signaling [154].

### Targeting the gut microbiome

Probiotics are living nonpathogenic microorganisms that help preserve the intestinal barrier, inhibit pathogen spread, minimize bacterial displacement, and prevent infection [155]. Nevertheless, genomic analysis in an epidemiological investigation detected six independent incidences of probiotic transfer from the capsule to the blood in ICU patients, resulting in bacteremia [156].

Prebiotics are indigestible dietary components, most of which comprise indigestible oligosaccharides that may specifically encourage the development and metabolism of beneficial gut microbiota, improving the balance of the intestinal flora and benefiting human health [157]. Cellulose supplementation has been associated with partly decreased systemic inflammation in mouse sepsis models, suggesting a survival benefit conferred by microbes [158].

Fecal microbial transplantation (FMT) can save mice from lethal sepsis caused by pathogens isolated from a septic patient, which is associated with spread of butyrate-producing Bacteroidetes, improvement in pathogen clearance, and restoration of host immunity via interferon regulatory factor 3 [159]. FMT may be an option for treating sepsis; however, donor screening is necessary to prevent spread of bacteria that may cause unfavorable infection. Moreover, it is important to consider the advantages and hazards of FMT in diverse patient groups.

An entirely different strategy is the use of absorbent materials to prevent enterogenic toxins and bacterial products from entering the blood circulation and the liver. Indeed, cation-exchange resin targeting hyperkalemia and hyperphosphatemia has shown efficacy [160]. Nonabsorbable nanopore carbon reduced portal vein pressure and liver biochemical markers in rats that underwent bile duct ligation, resulting in decreased endotoxin-induced KC stimulation [161, 162]. Therefore, by targeting the gut–liver axis, adsorbent materials is a possible treatment strategy for sepsis.

### High-density lipoprotein (HDL) as a potential therapy

One study revealed that HDL is able to neutralize LPS and accelerate LPS clearance via SR-BI-mediated LPS uptake in mice [163]. Numerous apolipoproteins (Apo) on HDL particles play crucial roles in clearing endotoxins to prevent infection.

For instance, the inflammatory response to LPS is inhibited when LPS binds to Apo AI or Apo E [164–166], but Apo AII and Apo CI bind to LPS, enhancing inflammation [167, 168]. In a study of 63 severe septic patients, HDL levels < 20 mg/dL and Apo AI < 100 mg/dL on day 1 were associated with an increase in overall and sepsis-attributable 30-day mortality rates, prolonged intensive care unit stay and hospital-acquired infection [169].

Moreover, reconstituted HDL infusion reduces endotoxin-induced histological tissue injury in the lung, liver, and intestines of rats [170]. In addition, 111-indium bacterial labeling in mice highlights the possibility of potential hepatic bacterial clearance promoted by HDL uptake [171]. Thus, HDL may be an important target for future sepsis prevention and therapy.

## Conclusions

During sepsis, gradual damage to gut barrier components due to inflammation causes increased intestinal permeability and intestinal dysbiosis, including pathogenic microbial overgrowth and translocation of abundant PAMPs and DAMPs to the liver via the portal circulation or biliary tract. Because of its physical proximity to the gut, the liver, a first-line immune organ acting as a gatekeeper between the gut and systemic circulation, can be rapidly shifted from immune hyporeactivity to produce a potent inflammatory response and effective adaptive immunity. However, an inappropriate immune response or overwhelming inflammation can be triggered by intestinal hyperpermeability and gut-derived PAMPs and DAMPs after sepsis, resulting in impaired hepatic clearance of pathogenic bacteria and metabolic disorder and contributing to MODS. Therefore, a better understanding of the gut–liver interaction in sepsis may help prevent and limit sepsis-induced liver damage and improve the prognosis of patients with sepsis.

## Data Availability

Not applicable.

## Abbreviations

AMPs: Antimicrobial peptides
AOAH: Acylhydrolase
Apo: Apolipoprotein
BPI: Bactericidal/permeability-increasing protein
CRIg: Complement receptor of the immunoglobulin superfamily
DAMPs: Damage-associated molecular patterns
FMT: Fecal microbial transplantation
HDL: High-density lipoprotein
IAPs: Intestinal alkaline phosphatase
IECs: Intestinal epithelial cells
IFN: Interferon
IL: Interleukin
KCs: Kupffer cells
LBP: LPS-binding protein
LPS: Lipopolysaccharide
LSECs: Liver sinusoidal endothelial cells
LTA: Lipoteichoic acid
MARCO: Macrophage receptor with collagen structure
MODS: Multiple organ dysfunction syndrome
MUCs: Mucins
MyD88: Myeloid differentiation factor 88
NETs: Neutrophil extracellular traps
NF-κB: Nuclear factor kappa-B
NK: Natural killer
NKT: Natural killer T
PAMPs: Pathogen-associated molecular patterns
Th1: T helper 1
TJs: Tight junctions
TLR: Toll-like receptor
TNF: Tumor necrosis factor
